# Surgical Emergencies, Together with the Emergencies Attendant on Parturition and the Treatment of Poisoning

**Published:** 1897-03

**Authors:** 


**Surgical Emergencies, together with the Emergencies attendant on Parturition and the Treatment of Poisoning.** By PAUL SWAIN. Fifth Edition. Pp. xviii., 254. London: J. & A. Churchill. 1896.

This new edition has been thoroughly brought up to date and well maintains the reputation of its predecessors. It would be better to devote more space to the use of the syphon method of emptying the stomach rather than to the use of the stomach pump (pp. 191—194), as the former is by far the more generally useful and the less risky. The book is essentially practical and thoroughly trustworthy.

